# An Unusual Laryngeal Foreign Body in Adult

**DOI:** 10.1155/2016/5798070

**Published:** 2016-11-23

**Authors:** Cire Ndiaye, Eric Joel Regonne, Houra Ahmed, Evelyne Siga Diom, Richard Edouard Alain Deguenonvo, Aminata Mbaye, Yilkal Zemene, Issa Cheikh Ndiaye

**Affiliations:** ^1^Department of Otolaryngology-Head and Neck Surgery, Fann Teaching Hospital, Dakar, Senegal; ^2^Department of Otolaryngology-Head and Neck Surgery, General Hospital of Grand Yoff, Dakar, Senegal; ^3^Department of Otolaryngology-Head and Neck Surgery, Mekelle University, Mek'ele, Ethiopia

## Abstract

The accidental aspiration of a foreign body is a frequent domestic accident among children but a rare occurrence in adults. The laryngeal impaction of a coin is an unusual accident; only a few cases have been reported in the literature. Diagnosis is mostly achieved by clinicoradiological examinations. The authors report an uncommon case of laryngeal impaction of a coin in a 21-year-old patient, presenting with dysphonia without dyspnea or stridor. The extraction was performed by endoscopy.

## 1. Introduction

The accidental aspiration of a foreign body (FB) is a frequent domestic accident among children but a rare occurrence in adults. An impacted laryngeal FB in children is not uncommon. It represents 44.83% of FB of respiratory tract in Senegal [1]. The laryngeal impaction of a coin is an unusual accident; at least 3 cases have been reported in the literature [2–4]. In all cases, hoarseness was the main complaint without respiratory disorder. Diagnosis was mostly achieved by clinicoradiological examinations. We report the case of a patient with an unusual impacted laryngeal foreign body and we discuss the diagnostic and therapeutic aspects.

## 2. Case Report

**Figure 1:**
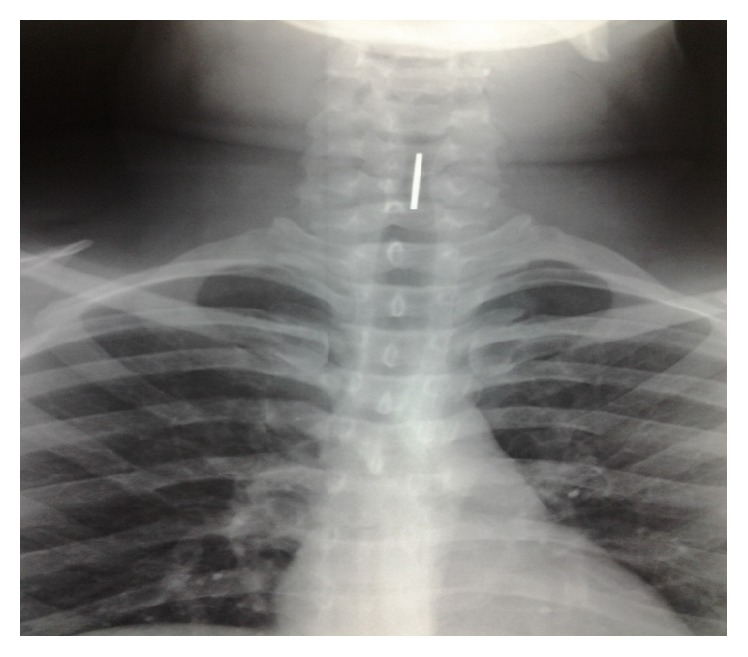
Frontal X-ray: anteroposterior view showing linear opacity from C4 to C5.

**Figure 2:**
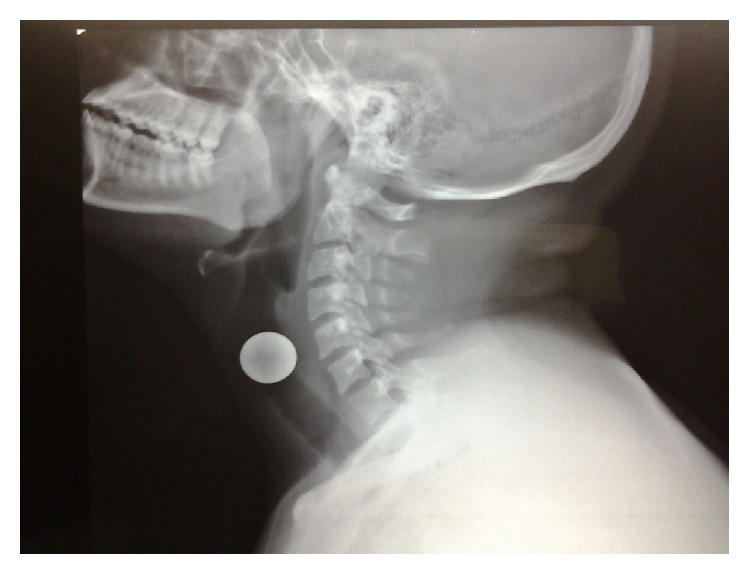
Lateral X-ray: lateral view showing rounded opacity in sagittal plane.

A 21-year-old patient, with no medical history, presented to our department with a sudden onset of change in his voice associated with a cough for 24 hours. The history revealed accidental ingestion of a coin that he was carrying in his mouth. Indirect laryngoscopy showed a metallic foreign body across the glottis. There were no dyspnea and no stridor. The clinical examination showed no other remarkable signs. X-ray of the neck (Figures 1 and 2) revealed a radiopaque foreign body, located in the laryngeal area, facing C5.

**Figure 3:**
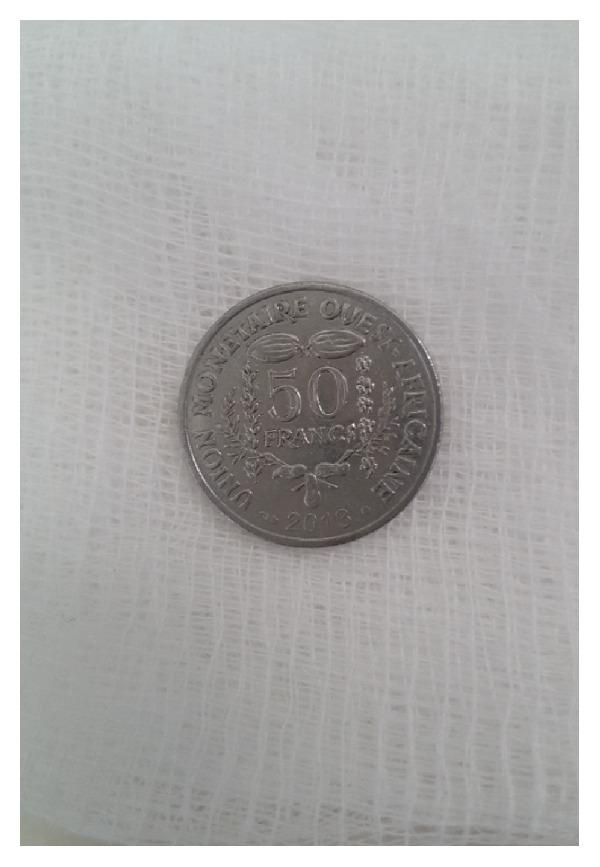
The removed coin.

We have previously explained to the patient that a tracheotomy could be performed postoperatively if he had a respiratory distress. A direct laryngoscopy was performed under general anesthesia and spontaneous ventilation. The coin was in sagittal position, visible through the glottis. The foreign body was extracted and it was a 50 CFA Francs metallic coin (with 22 mm diameter) (Figure 3). The glottis and subglottis showed no evidence of inflammation. No tracheotomy was performed after the removal. Postendoscopic period was uneventful. The patient was discharged the next day following the procedure. In follow-up, patient had regained his voice.

## 3. Discussion

Foreign body impaction in larynx in a child is not uncommon. It represents 44.83% of FB of respiratory tract in Senegal [1]. Its occurrence is rare among adults, affecting adults who are unable to protect the airway such as those mentally impaired, under alcoholism, and elderly and persons with psychoses and neurological disorders [2, 5, 6].

The foreign bodies found are most usually of an organic, food, or bone nature [7]. The presence of a coin in the larynx is a rare situation. Most cases reported in the literature are cases report [2–4]. Clinically, dysphonia and cough are the most consistent symptoms [2, 3]. Despite the size of a coin, dyspnea has never been reported. Hada et al. [2] explain the absence of respiratory distress by the sagittal position of the coin causing only a partial obstruction. The same authors mentioned as well the less inflammatory character of inorganic foreign bodies compared to organic bodies. The lack of symptoms is the reason for the delay in the consultation. Hathiram et al. [3] reported a 3-day consultation delay; it was just 1 day in our case.

Opacity on lateral X-ray, around C4-C5 found in our patient, is present in all published cases. This could be explained by the fact that the coin can only access the glottal slit when it is disposed in the sagittal plane (Figure 2), unlike esophageal foreign body accidents, where the coin is present in the frontal plane.

The treatment options depend on the habits of each ENT team and conditions of practice. Hathiram et al. [3] performed an elective tracheotomy before extracting the FB, to protect airways. The coin was removed under general anesthesia. Singh et al. [4] removed the coin under local anesthesia and sedation, while Hada et al. [2] performed an extraction of the FB under general anesthesia. In all cases, coins were removed by direct laryngoscopy. We preferred the option of general anesthesia with spontaneous ventilation. This anesthetic technique has the advantage of reducing the length of hospital stay. The risk of laryngeal spasm is low in adults.

## 4. Conclusion

The presence of a coin in the respiratory tract is an exceptional occurrence. Clinical signs are often insignificant and routine. The rule prohibiting children from carrying objects in the mouth also prevails among adults.
